# Beyond the Blue Zones: Healthy Aging and Extreme Longevity in Italy (1982–2025)—An Ecological Analysis of Demographic, Metabolic, and Nutritional Correlates

**DOI:** 10.3390/nu18121952

**Published:** 2026-06-17

**Authors:** Silvana Mirella Aliberti, Daria Nurzynska, Mario Capunzo

**Affiliations:** 1 Department of Medicine, Surgery and Dentistry “Scuola Medica Salernitana”, University of Salerno, 84081 Baronissi, Italy; dnurzynska@unisa.it (D.N.); mcapunzo@unisa.it (M.C.); 2 Division of Preventive Medicine, Dresden International University (DIU), 01067 Dresden, Germany; 3 Complex Operational Unit of Health and Hygiene, University Hospital “San Giovanni di Dio e Ruggi d’Aragona”, 84131 Salerno, Italy

**Keywords:** extreme longevity, healthy aging, centenarians, NCD mortality, Mediterranean diet, regional disparities, Cilento, Italy

## Abstract

**Background/Objectives**: Italy is among the countries with the highest life expectancy and extreme longevity worldwide, yet marked regional disparities persist. This nationwide ecological study examined temporal trends in population aging and extreme longevity across the 20 Italian regions (1982–2025) and explored regional correlates non-communicable disease (NCD) mortality and contemporary behavioral/nutritional indicators, with attention to emerging southern hotspots such as Cilento. **Methods**: Longevity indicators (Aging Tendency, Longevity Index [LI%], Centenarity Index [CI%], 85+ and 90+ ratios) were derived from ISTAT demographic data. Age-standardized mortality rates for five major NCDs (1990–2023) were obtained from WHO HFA-DB. Behavioral and nutritional indicators for adults aged ≥65 years (2024) were extracted from HFA-Italy. Regional associations were assessed using Spearman correlations within an ecological, hypothesis-generating framework. **Results**: All longevity indicators increased steadily from 1982 to 2025, with northern and central regions showing the highest values. Lower long-term mortality from diabetes mellitus and cerebrovascular diseases showed the strongest regional correlations with higher LI% and CI%. Nutritional profiles were generally more favorable in northern regions. The Cilento area emerged as a notable southern hotspot, displaying longevity indicators comparable to Sardinia and above the regional average. **Conclusions**: Regional patterns of extreme longevity in Italy reflect the interplay of demographic dynamics, NCD mortality burden, and contemporary lifestyle profiles. While northern regions maintain a clear advantage, specific southern areas such as Cilento demonstrate that favorable longevity outcomes can emerge in diverse macro-regional contexts. These findings highlight the value of regionally tailored strategies to promote healthy aging and reduce geographical disparities.

## 1. Introduction

### 1.1. Global Population Aging Context and the Rise in Extreme Longevity

Population aging is one of the most profound demographic transformations of the 21st century and represents a major challenge for public health, social systems, and economic sustainability worldwide. According to the United Nations World Population Prospects (WPP) 2024, the global population aged ≥65 years is projected to more than double by 2050, driven by sustained fertility decline, improved survival, and the aging of large post-war cohorts [1,2,3,4,5]. Within this broader transition, the rapid increase in the number of centenarians has emerged as a key demographic marker of extreme longevity, reflecting both improved survival and changing trajectories of aging [1,6,7,8,9,10,11,12,13,14,15].

### 1.2. Longevity in Europe and Italy: A Context of Marked Regional Heterogeneity

Europe is among the world regions with the highest proportion of adults aged ≥65 years, reflecting one of the most advanced stages of population aging globally. South Korea has also emerged as a leading example of rapid demographic aging, having reached “super-aged” status even faster than Japan. Several countries—including France, Italy, and Spain—rank among the world leaders in life expectancy and centenarian prevalence [1,16,17]. France counted approximately 30,000 centenarians in 2024 [16,17], while Italy counted 23,548 centenarians as of 1 January 2025 [18]. National life expectancy remains among the highest in Europe, although with substantial geographic variation by sex and region [11,12,13,19,20].

This heterogeneity has long attracted scientific interest, with some areas—such as Sardinia and parts of southern Italy—identified as regions with a high concentration of long-lived individuals [21,22,23,24,25]. These areas are often described as “Blue Zones”, that is, geographical regions characterized by an exceptionally high prevalence of centenarians and shared lifestyle and environmental features linked to exceptional longevity. Within this framework, the Cilento area has also been highlighted in previous investigations as an emerging Italian longevity hotspot [8,9,24,25].

However, beyond these well-known hotspots, Italy as a whole represents a valuable setting for examining regional differences in longevity, given its diverse demographic, socioeconomic, and epidemiological profiles.

### 1.3. Aging, Non-Communicable Diseases, and Regional Epidemiological Profiles

The increase in survival into advanced age is closely linked to the long-term burden of non-communicable diseases (NCDs), including cardiovascular diseases, cancers, diabetes mellitus, chronic respiratory diseases, and cerebrovascular conditions, which remain the leading causes of morbidity and mortality globally and in Europe [26]. In Italy, the epidemiological transition toward chronic degenerative diseases has contributed to rising life expectancy, yet substantial regional disparities persist in NCD mortality patterns [19,20].

Understanding how long-term mortality trends intersect with regional longevity indicators is essential for characterizing population aging trajectories. Regions with exceptionally high proportions of older adults or centenarians often display distinct historical patterns of cardiovascular, metabolic, or cerebrovascular mortality, which may provide useful context for understanding regional trajectories without implying direct causal relationships.

### 1.4. Regional Longevity as a Multidimensional Phenomenon

Longevity is shaped by the interplay of demographic, environmental, behavioral, and social factors operating across the life course [6,7,8,9,10,11,12,13,14,15,21,22,23,24,25,26,27,28,29,30,31,32,33]. While individual-level determinants cannot be assessed through ecological designs, regional analyses can provide valuable insights into contextual patterns associated with population aging. In this perspective, integrating demographic indicators, long-term mortality trends, and contemporary behavioral and nutritional data may help characterize the broader environments in which longevity emerges, without implying causal mechanisms.

### 1.5. Rationale and Objectives

Despite substantial interest in longevity and demographic aging, few studies have provided an integrated, long-term ecological assessment of regional longevity patterns and NCD mortality across all Italian regions. Existing research has often focused on specific hotspots or on national-level life expectancy, with limited comparative analyses spanning multiple decades and incorporating diverse indicators.

To address this gap, the present study offers a comprehensive ecological overview of regional longevity in Italy by integrating:

- Standardized indicators of extreme longevity and population aging (1982–2025);
- Age-standardized mortality trends for major NCDs (1990–2023);
- Selected behavioral and nutritional indicators among adults aged ≥65 years (2024).

The objectives of this study are to:

1. Describe regional patterns and temporal trends in population aging and extreme longevity indicators across Italy from 1982 to 2025.
2. Analyze long-term regional trends in age-standardized mortality for major non-communicable diseases from 1990 to 2023.
3. Contextualize regional longevity profiles using contemporary behavioral and nutritional indicators in older adults.

This ecological, descriptive approach aims to provide an updated and multidimensional characterization of regional longevity heterogeneity in one of the world’s longest-lived populations, offering a foundation for future analytical and causal investigations.

## 2. Materials and Methods

### 2.1. Study Design

This study employed a regional ecological observational design integrating descriptive temporal analyses, comparative geographic assessments, and exploratory correlational analyses to investigate patterns of extreme longevity and their associations with non-communicable disease (NCD) mortality across Italy’s 20 administrative regions. The aim was to characterize long-term trends in population aging and extreme longevity from 1982 to 2025, examine historical and projected patterns of major non-communicable disease (NCD) mortality, and explore ecological associations between longevity indicators and NCD mortality burdens, as well as selected behavioral and nutritional factors (updated to 2024).

Given the ecological framework, all analyses were conducted using aggregated regional or macro-regional population-level data. Findings are therefore intended to identify broad epidemiological patterns, generate hypotheses, and inform public health strategies, rather than establish causal relationships at the individual level. Extreme longevity indicators (LI%, CI%, 85+ and 90+ ratios) represent demographic survival patterns and do not capture individual-level functional health or clinical dimensions of healthy aging.

### 2.2. Data Sources

#### 2.2.1. Demographic Data

Demographic data were obtained from the Italian National Institute of Statistics (ISTAT) [34]. Population counts by sex, region, and age group (≥65, ≥85, ≥90, and ≥100 years) were extracted for four benchmark periods: 1982 (Intercensal Reconstruction), 2001 (National Census), 2019 (Demographic Statistics), and 2025 (official population estimates as of 1 January 2025). These time points were selected to capture long-term demographic transitions while providing both historical depth and contemporary relevance.

#### 2.2.2. Mortality Data

The primary source for age-standardized mortality rates (ASMRs) was the World Health Organization European Health for All database (HFA-DB) [35]. Data on five major non-communicable diseases—neoplasms, ischemic heart disease, cerebrovascular disease, diabetes mellitus, and chronic lower respiratory diseases—were extracted for the years 1990, 2001, 2011, 2023. Choropleth maps were generated directly from the HFA-DB online platform using standardized parameters and the European Standard Population. Analyses were performed at both national and macro-regional levels (North-West, North-East, Centre, South, and Islands), with available projections up to 2030. Projected values correspond to the official forecast series provided directly by the WHO HFA-DB, which applies its own standardized projection algorithms. No additional modelling or extrapolation by the authors. Age-standardized rates were used for regional choropleth maps and temporal trend analyses to allow fair comparisons across regions with different age structures.

Supplementary cause-specific mortality data for 2023 were retrieved from the ISTAT Esploradati platform [36] and used to calculate age-specific mortality rates (population aged ≥65 years) at the macro-regional level. These data are presented in a dedicated table to provide greater age-specific detail complementary to standardized rates from HFA-DB. Additionally, total mortality data for the population aged ≥65 years and ≥90 years in 2025 were obtained from the ISTAT Demo platform at the regional level [37]. Crude mortality rates (per 100,000) were calculated for these 2025 data to reflect the actual mortality burden on the current older population structure, enabling direct comparison with the 2025 longevity indicators. These mortality rates are expressed per 100,000 individuals within each age group (≥65 and ≥90). Regions with smaller older populations may therefore show higher rate variability due to denominator effects, a known characteristic of crude age-specific mortality calculations.

#### 2.2.3. Behavioral and Nutritional Indicators

Behavioral and nutritional indicators for adults aged ≥65 years were obtained from HFA-Italy database (2024) [38], including regular vegetable consumption, fish consumption, bovine meat consumption, adequate breakfast habits, and overweight/obesity prevalence. These indicators originate from nationally representative population surveys conducted by ISTAT and the Italian Ministry of Health and are harmonized by WHO for inclusion in HFA-Italy. Most variables are survey-based and self-reported, as they derive from structured questionnaires administered to adult respondents. Adequate breakfast was defined according to national surveillance standards as the consumption of a morning meal including at least one solid or liquid food item. Fish consumption followed the operational definition used in HFA-Italy and included all fish products, encompassing both finfish and seafood (e.g., mollusks and crustaceans). These variables were included as proxies for modifiable lifestyle factors potentially linked to healthy aging.

Geographic distributions were visualized using choropleth maps at the regional level. Maps were generated directly from the HFA-Italy database online platform. Additionally, bar charts at the macro-area level were created to allow simultaneous comparison of all six indicators across the five Italian macro-areas (North-West, North-East, Centre, South, and Islands). Behavioral and nutritional indicators from HFA-Italy are provided as standardized regional aggregates derived from nationally representative surveys. These data are therefore directly comparable with the regional mortality and longevity indicators used in this study. The aim of the analysis was to examine differences across Italian regions, not within-region variability; therefore, the use of region-level aggregates is appropriate for the ecological design adopted.

### 2.3. Definition of Longevity Indicators

Longevity indicators were defined based on previously validated approaches [39,40,41,42,43], ensuring methodological consistency across the author’s research:

- Aging Tendency: proportion of individuals aged ≥65 years relative to the total population.
- The 85+ ratio: proportion of individuals aged ≥85 years in the total population.
- The 90+ ratio: proportion of individuals aged ≥90 years in the total population.
- Longevity Index (LI%) = (population aged ≥90 years/population aged ≥65 years) × 100.
- Centenarity Index (CI%) = (population aged ≥100 years/population aged ≥90 years) × 100.
- Female-to-male (F/M) ratio: ratio of females to males among nonagenarians (≥90 years) and centenarians (≥100 years), used as a demographic measure of sex difference in survival at advanced ages rather than as direct indicator of longevity.

These indicators were chosen to capture complementary aspects of population aging and extreme longevity.

### 2.4. Statistical Analysis

Descriptive analyses were conducted to summarize temporal trends and regional variations in demographic aging and longevity indicators across the four benchmark years (1982, 2001, 2019, and 2025). Spatial distributions of NCD mortality were assessed using choropleth maps for the years 1990, 2001, 2011, and 2023, extracted directly from the WHO HFA-DB platform. Temporal trends were examined using macro-regional line charts (North-West, North-East, Centre, South, and Islands).

Exploratory ecological associations between 2025 longevity indicators (LI%, CI%, Aging Tendency and F/M ratio) and both NCD mortality rates and 2024 behavioral/nutritional indicators were assessed using Spearman’s rank correlations across the 20 Italian regions.

To address the small sample size (*n* = 20) and enhance robustness, the following procedures were implemented:

- Correction for multiple comparisons using the Benjamini–Hochberg false discovery rate (FDR) method.
- Bootstrap bias-corrected accelerated (BCa) 95% confidence intervals with 10,000 replications for the main correlations.
- Leave-one-out (LOO) sensitivity analysis, repeating the correlations after removing one region at a time, to evaluate stability and influence of individual observations.
- Sensitivity analyses using different mortality time windows, including cause-specific mortality rates for 2023, crude total mortality rates for 2025 (65+ and 90+), and historical averages (1991–2021).
- Exploratory multivariate linear regression models were performed on selected variables to assess potential independent associations.

Given the ecological design and limited number of units (*n* = 20), all results are interpreted as exploratory associations rather than causal determinants. Behavioral and nutritional indicators from 2024 were included as contemporary contextual measures of the regional environment and are not intended to represent early-life exposures of centenarian cohorts. Statistical significance was set at q < 0.05 after FDR correction (two-tailed tests). All analyses were conducted using STATA version 16.1 (StataCorp., College Station, TX, USA) [44].

### 2.5. Ethical Considerations

This study used exclusively publicly available, aggregated population-level data from official databases (ISTAT and WHO). No identifiable individual data were involved; therefore, ethical approval was not required.

## 3. Results

### 3.1. Demographic Aging Trends in Italian Regions (1982–2025)

**Figure 1:**
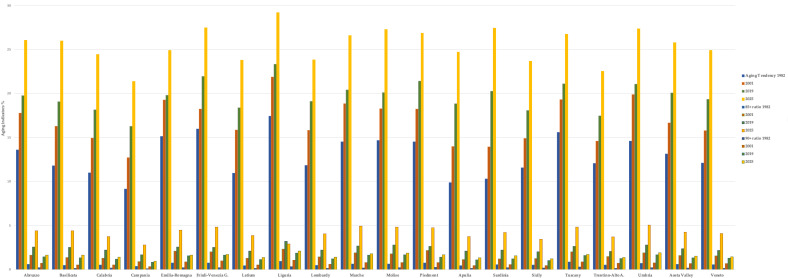
Grouped bar chart illustrating regional patterns in key aging indicators across the 20 Italian regions at four time points (1982, 2001, 2019, 2025). The chart reports the Aging Tendency, the proportion of individuals aged ≥85 years, and the proportion of individuals aged ≥90 years. Higher values indicate more advanced demographic aging. Northern and central regions consistently show higher levels across all indicators compared with southern regions. Data: ISTAT.

The demographic analysis, based on four key time points—Intercensal Reconstruction (1982), National Census (2001), Demographic Statistics (2019), and official data as of 1 January 2025—documents a continuous and regionally heterogeneous aging process across Italy. A steady rise in the proportion of the population aged ≥65 years, together with increasing Longevity Index (LI%) and Centenarian Index (CI%), confirms a marked expansion of both the older and oldest-old population (Figure 1).

#### 3.1.1. Aging Trends and Regional Variability

As shown in the radial chart (Figure 1), all three indicators—aging tendency, 85+ ratio, and 90+ ratio—exhibited a clear upward trajectory over the 43-year period. Northern and central regions consistently displayed the most advanced aging profiles. In 2025, Liguria recorded the highest Aging Index, followed by Emilia-Romagna, Tuscany, and Friuli-Venezia Giulia. In contrast, southern regions such as Campania and Calabria maintained comparatively lower values, reflecting a slower pace of demographic aging.

#### 3.1.2. Growth of the Oldest-Old Population

The expansion of the oldest-old segments was particularly pronounced. The proportion of individuals aged ≥85 and ≥90 years increased substantially across the country between 1982 and 2025. Liguria, Marche, and Molise showed among the largest gains in the 85+ population, while Liguria, Tuscany, and Emilia-Romagna, and Trentino-Alto Adige recorded the highest proportions of individuals aged ≥90 years in 2025 (Figure 1).

Notably, the outward shift of the 2025 line (yellow) across most regions in the radial plot visually confirms the progressive aging of the Italian population and the growing representation of individuals reaching advanced old age. These trends were accompanied by marked regional disparities, with northern and central Italy generally exhibiting higher values across all indicators compared to the South, although some southern regions showed notable increases in specific longevity metrics.

#### 3.1.3. Centenarity Index (CI%)

The Centenarian Index (CI%), defined as the ratio of individuals ≥100 years to those aged ≥90 years, increased across all regions between 1982 and 2025. By 2025, the highest values were observed in Molise (3.32), Calabria (3.11), and Friuli-Venezia Giulia (2.93), indicating relatively higher proportions of individuals reaching extreme old age compared with earlier decades (Table 1). Given the small population sizes of regions such Molise and Basilicata, CI% values in these areas may show greater year-to-year variability, reflecting statistical instability rather than substantive demographic differences.

#### 3.1.4. Longevity Index (LI%) and Regional Differences

The Longevity Index (LI%), calculated as the ratio of individuals aged ≥90 years to those aged ≥65 years, showed a consistent increase nationwide, with marked regional variation. In 2025, the highest values were recorded in Liguria (7.23), Emilia-Romagna (6.67) and Friuli-Venezia Giulia (6.35), whereas Campania (4.54) and Sicily (5.17) showed comparatively lower values (Table 2).

#### 3.1.5. Gender Differences in Aging (F/M Ratio)

A pronounced female survival advantage was observed across all regions and time points. In 2025, the highest female-to-male ratios among centenarians were recorded in Valle d’Aosta (12.67), Lombardy (6.93), and Friuli-Venezia Giulia (6.46), while the lowest values were found in Calabria (2.91) and Basilicata (3.26). A similar, though less pronounced, pattern was observed among nonagenarians (Table 2).

#### 3.1.6. Total Mortality Rates in the Older Population (2025)

Total mortality rates in the population aged ≥65 years showed substantial regional variation in 2025. The lowest rates were observed in several northern and north-eastern regions, such as Lombardy (4026 per 100,000), Trentino-Alto Adige (4036), and Veneto (8088). In contrast, markedly higher rates were recorded in southern regions, particularly Basilicata (67,672), Calabria (21,341), and Molise (12,624).

Among individuals aged ≥90 years, mortality rates were higher and displayed greater heterogeneity, with the highest values in Molise (855,830 per 100,000), Basilicata (381,660), and Abruzzo (167,730), and the lowest in Trentino-Alto Adige (25,445) (Table 3).

### 3.2. Historical and Projected NCD Mortality Trends (1990–2030)

The analysis of five major non-communicable diseases (neoplasms, ischemic heart diseases, cerebrovascular diseases, diabetes mellitus, and chronic lower respiratory diseases) in the population aged ≥65 years showed a general decline in age-standardized mortality rates from 1990 to 2023, together with persistent regional differences.

#### 3.2.1. Spatial Patterns of NCD Mortality (1990–2023)

**Figure 2:**
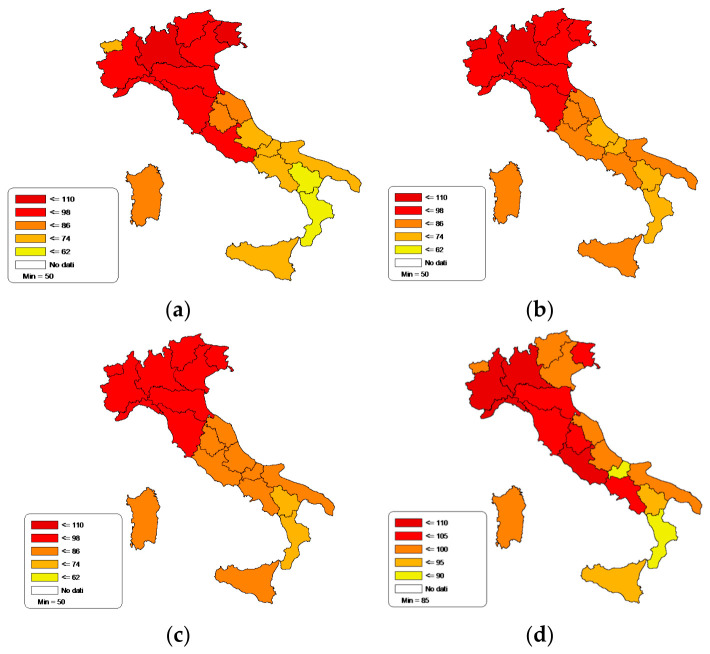
Choropleth map of age-standardized mortality rates from neoplasms among individuals aged ≥65 years in Italian regions: (**a**) 1990; (**b**) 2001; (**c**) 2011; (**d**) 2023. Maps were generated directly from the WHO European Health for All Database (HFA-DB)/HFA-Italy online platform. ‘No dati’ indicates regions with no available data.

**Figure 3:**
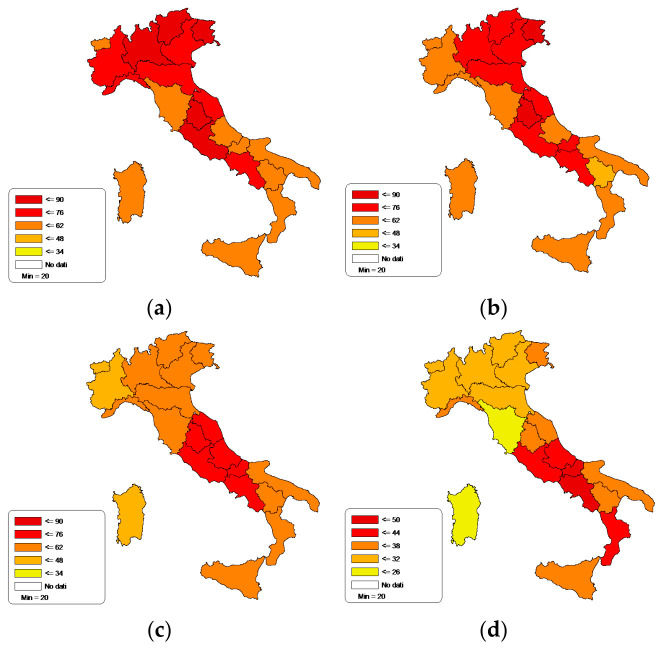
Choropleth map of age-standardized mortality rates from ischemic heart diseases among individuals aged ≥65 years in Italian regions: (**a**) 1990; (**b**) 2001; (**c**) 2011; (**d**) 2023. Maps were generated directly from the WHO European Health for All Database (HFA-DB)/HFA-Italy online platform. ‘No dati’ indicates regions with no available data.

**Figure 4:**
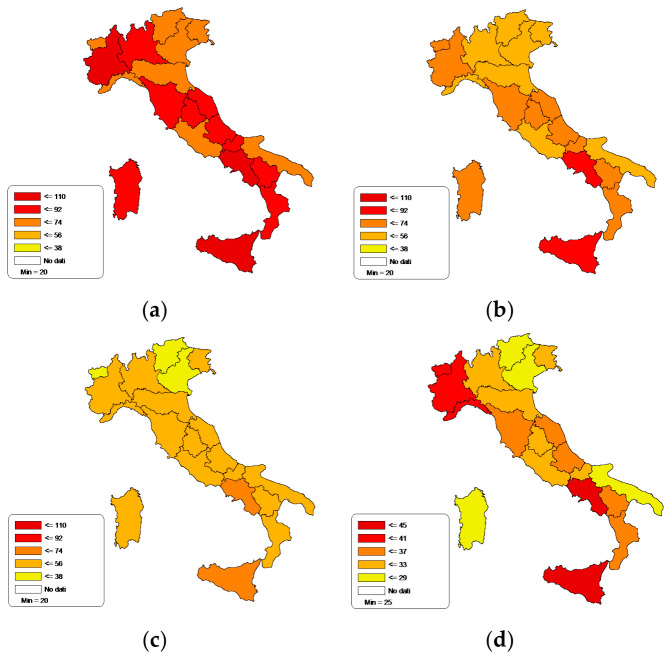
Choropleth map of age-standardized mortality rates from cerebrovascular diseases among individuals aged ≥65 years in Italian regions: (**a**) 1990; (**b**) 2001; (**c**) 2011; (**d**) 2023. Maps were generated directly from the WHO European Health for All Database (HFA-DB)/HFA-Italy online platform. ‘No dati’ indicates regions with no available data.

**Figure 5:**
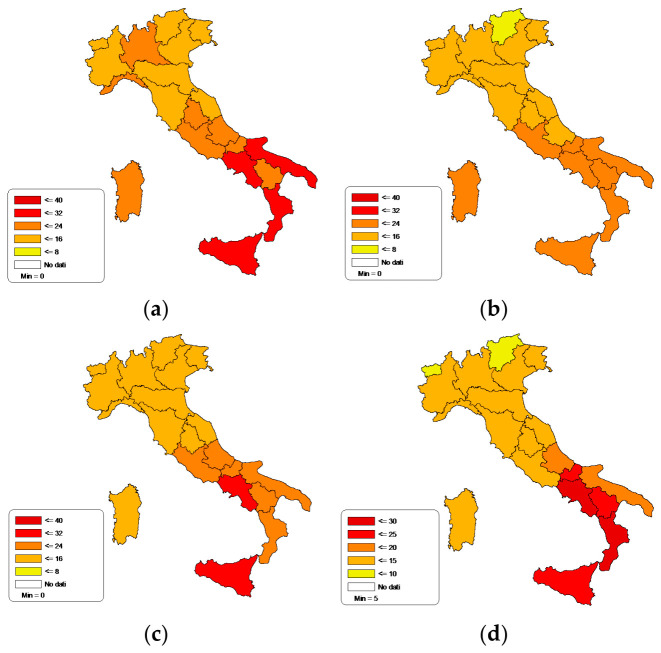
Choropleth map of age-standardized mortality rates from diabetes mellitus among individuals aged ≥65 years in Italian regions: (**a**) 1990; (**b**) 2001; (**c**) 2011; (**d**) 2023. Maps were generated directly from the WHO European Health for All Database (HFA-DB)/HFA-Italy online platform. ‘No dati’ indicates regions with no available data.

**Figure 6:**
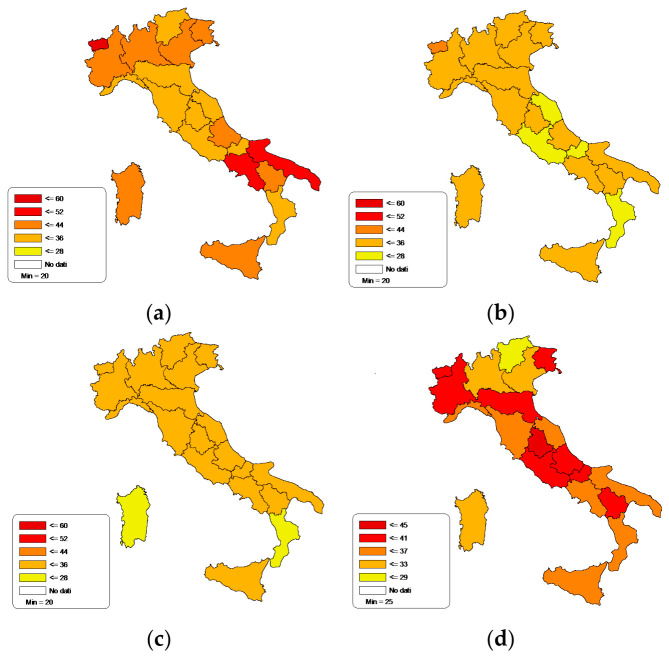
Choropleth map of age-standardized mortality rates from chronic lower respiratory diseases among individuals aged ≥65 years in Italian regions: (**a**) 1990; (**b**) 2001; (**c**) 2011; (**d**) 2023. Maps were generated directly from the WHO European Health for All Database (HFA-DB)/HFA-Italy online platform. ‘No dati’ indicates regions with no available data.

Choropleth maps of age-standardized mortality rates (per 100,000 inhabitants) for the five major non-communicable diseases in the population aged ≥65 years are presented in Figure 2, Figure 3, Figure 4, Figure 5 and Figure 6.

For neoplasms, in 1990 the highest rates were recorded in northern and central regions, with several areas (Piedmont, Lombardy, Veneto, Emilia-Romagna, Liguria) exceeding 35–40 deaths per 100,000, while southern regions generally remained below 25. The national average was 26.07 in 1990. By 2001 the national rate had risen to 118.17, then declined to 113.39 in 2011, reaching 101.12 in 2023. Northern regions consistently showed higher rates than southern regions across all time points.

For cerebrovascular diseases, in 1990 southern regions and the Islands recorded the highest mortality rates (often above 90–110 per 100,000), with a national average of 78.53. Mortality decreased over time, reaching 57.92 in 2001, 46.66 in 2011, and 33.21 in 2023. Southern regions (particularly Calabria, Puglia, and Sicily) maintained relatively higher values than northern regions throughout the period.

For diabetes mellitus, a marked North–South gradient was observed. In 1990, southern regions showed higher mortality, with Calabria, Puglia, Campania, Sicily, and Sardinia in the highest categories (above 30 per 100,000), compared with a national average of 19.89. Rates declined to 15.19 in 2001 and 15.74 in 2011, reaching 15.2 in 2023. The geographical gradient remained evident across all years.

For ischemic heart diseases, higher rates were recorded in northern and central regions in 1990 (national average 68.88). Mortality declined to 60.7 in 2001, 55.26 in 2011, and 33.74 in 2023. By 2023 the North–South difference had narrowed, although regional variability persisted.

For chronic lower respiratory diseases, the national average was 37.91 in 1990, with relatively higher rates in certain northern and central areas. Mortality decreased to 29.84 in 2001, remained around 31.02 in 2011, and reached 35.37 in 2023, with a more heterogeneous regional distribution compared to the other NCDs.

In summary, all five NCDs showed an overall decline in age-standardized mortality rates between 1990 and 2023. The magnitude and geographical pattern of this decline varied by cause: neoplasms and ischemic heart diseases tended to be higher values in northern and central regions in the earlier years, whereas diabetes mellitus and cerebrovascular diseases consistently showed higher values in southern regions and the Islands across the entire study period.

Age-specific mortality rates for the five major non-communicable diseases in the population aged ≥65 years are presented by macro-area in Table 4. The South and Islands recorded the highest rates for diabetes mellitus (195.07 and 201.07 per 100,000, respectively). The Centre showed the highest rates for ischemic heart diseases (314.07), cerebrovascular diseases (305.07), and chronic lower respiratory diseases (398.07). The North-East also showed high values for chronic lower respiratory diseases (334.07). For neoplasms, the highest rates were observed in the Centre (99.85), followed by the North-East (98.07) and North-West (94.18). These data indicate a persistent North–South gradient for diabetes mellitus, while other causes display more heterogeneous regional distributions, with several conditions reaching their highest values in the Centre and North-East macro-areas (Table 4).

#### 3.2.2. Temporal Trends and Projections (1990–2030)

**Figure 7:**
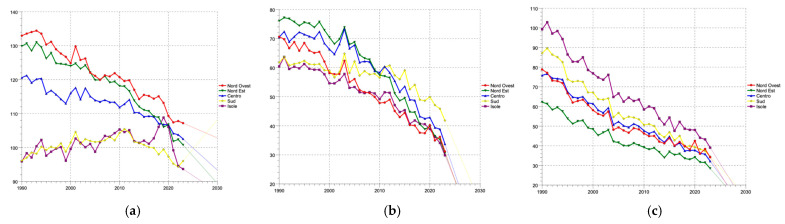
Temporal trends and projections of age-standardized mortality rates (per 100,000) for neoplasms, ischemic heart disease, and cerebrovascular disease among individuals aged ≥65 years by Italian macro-area, 1990–2030. (**a**) Neoplasms, (**b**) ischemic heart disease, (**c**) cerebrovascular disease. Solid lines represent observed data; dashed lines represent projections. Data source: WHO European Health for All Database (HFA-DB).

**Figure 8:**
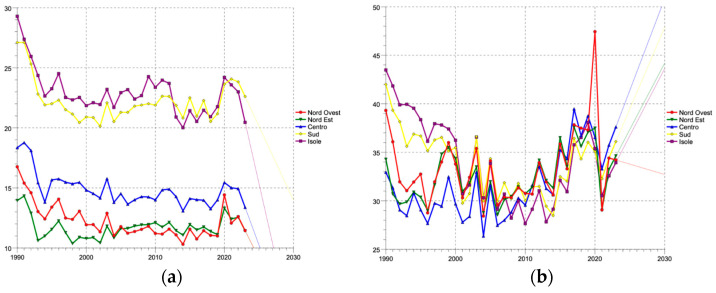
Temporal trends and projections of age-standardized mortality rates (per 100,000) for diabetes mellitus and chronic lower respiratory diseases among individuals aged ≥65 years by Italian macro-area, 1990–2030. (**a**) Diabetes mellitus, (**b**) chronic lower respiratory diseases. Solid lines represent observed data; dashed lines represent projections. Data source: WHO European Health for All Database (HFA-DB).

Temporal trends and projections (1990–2030) of age-standardized mortality rates for the major non-communicable diseases in the population aged ≥65 years, by Italian macro-area (North-West, North-East, Centre, South, and Islands), are presented in Figure 7 and Figure 8.

All five NCDs showed a clear overall declining trend from 1990 to 2023, although the magnitude and stating levels differed substantially by cause and macro-area. Neoplasms (Figure 7a) exhibited a marked decline in all macro-areas, with the North-West and North-East starting from higher levels. Ischemic heart diseases (Figure 7b) showed a steep and consistent reduction across the country, particularly pronounced in the northern regions where baseline rates were highest. Cerebrovascular disease (Figure 7c) also declined substantially, with southern regions and the Islands maintaining relatively higher rates throughout most of the period. Diabetes mellitus (Figure 8a) displayed a more heterogeneous pattern, with the South and Islands showing persistently higher mortality compared to northern macro-areas. Chronic lower respiratory diseases (Figure 8b) showed a general downward trend interrupted by a sharp peak between 2020–2022, likely attributable to the impact of the COVID-19 pandemic, followed by a resumption of declining trajectory.

Projections to 2030 suggest a continuation of the overall decline for most causes. These projected values correspond to the official forecast series provided directly by the WHO HFA-DB and were not modelled independently by the authors. However, regional disparities are expected to persist, with slower rates of improvement anticipated in the South and Islands, particularly for diabetes mellitus.

#### 3.2.3. Behavioral and Nutritional Indicators in the Population Aged ≥65 Years (2024)

**Figure 9:**
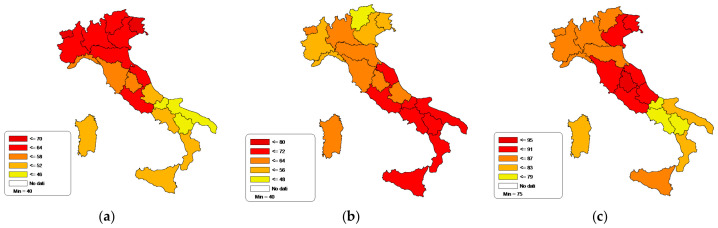
Choropleth maps showing the prevalence of healthy behavioral and nutritional indicators among individuals aged ≥65 years by Italian region, 2024. (**a**) Regular vegetable consumption (at least once per day); (**b**) fish consumption (several times per week); (**c**) adequate breakfast habits. Maps were generated directly from the WHO European Health for All Database (HFA-DB)/HFA-Italy online platform. ‘No dati’ indicates regions with no available data.

**Figure 10:**
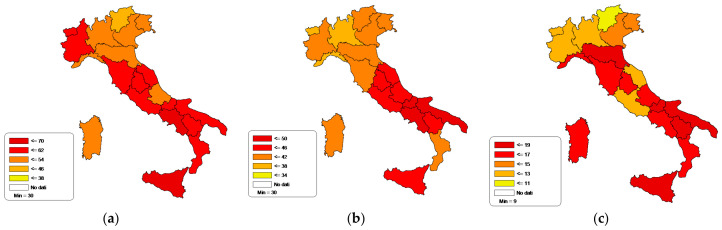
Choropleth maps showing the prevalence of bovine meat consumption, overweight and obesity among individuals aged ≥65 years by Italian region, 2024. (**a**) Bovine meat consumption (several times per week); (**b**) overweight prevalence (BMI ≥ 25 kg/m^2^); (**c**) obesity prevalence (BMI ≥ 30 kg/m^2^). Maps were generated directly from the WHO European Health for All Database (HFA-DB)/HFA-Italy online platform. ‘No dati’ indicates regions with no available data.

**Figure 11:**
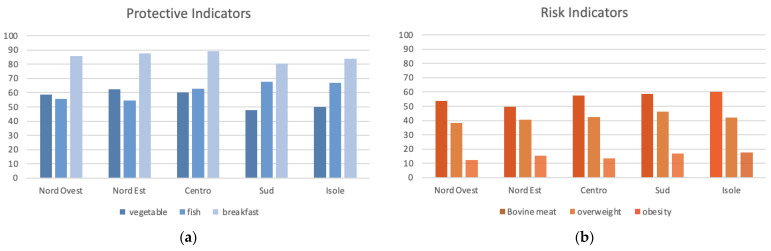
Prevalence of behavioral and nutritional indicators among individuals aged ≥65 years by Italian macro-areas, 2024. (**a**) Protective indicators: regular vegetable consumption (at least once per day), fish consumption (several times per week), and adequate breakfast habits. (**b**) Risk indicators: bovine meat consumption (several times per week), overweight prevalence (BMI ≥ 25 kg/m^2^), and obesity prevalence (BMI ≥ 30 kg/m^2^). Data source: HFA-Italy database.

Selected behavioral and nutritional indicators in the population aged ≥65 years were examined using data from the Italian adaptation of the WHO Health for All database (HFA-Italy) for the year 2024 (Figure 9, Figure 10 and Figure 11).

Choropleth maps at the regional level (Figure 9 and Figure 10) revealed a clear North–South gradient across most indicators. The percentage of individuals consuming vegetables at least once a day was generally higher in northern and central regions (ranging from 57% to 69%, with the highest values in Valle d’Aosta 64.67%, Veneto 62.94%, and Friuli-Venezia Giulia 69.47%) compared to southern regions and the Islands (ranging from 41% to 55%, with the lowest values in Molise 48.45%, Campania 49.25%, and Sicily 49.28%).

Similarly, the proportion reporting adequate breakfast was higher in the North and Centre (generally between 84% and 89%, with peaks in Lazio 88.92%, and March 87.64%) than in several southern regions (ranging from 78% to 84%).

Consumption of fish several times a week showed a less clear geographical pattern, with relatively higher frequencies in some central and southern regions (e.g., Marche 62.19%, Campania 70.75%, and Basilicata 65.96%) and more variable values in the North.

Consumption of bovine meat several times a week ranged from 39% to 64% across regions, without a strong consistent gradient.

Overweight and obesity displayed a marked North–South gradient. The prevalence of overweight (BMI ≥ 25) was substantially higher in southern regions (reaching 47.86% in Campania and 47.68% in Molise) to the northern regions (generally between 34% and 42%). Obesity prevalence followed a similar pattern, with higher values in the South and Islands (typically 16–19%) compared to the North (generally 9–14%, with the lowest in Trentino-Alto Adige 9.64%).

A complementary bar chart at the macro-area level (Figure 11) confirmed these patterns, highlighting the overall better profile of protective indicators (vegetable consumption, fish consumption, and adequate breakfast habits) in the northern macro-areas and the higher prevalence of risk indicators (bovine meat consumption, overweight, and obesity) in the southern macro-areas and the Islands.

Overall, these 2024 data indicate that northern and central regions generally showed better profiles for daily vegetable consumption and regular breakfast habits, while southern regions exhibited higher prevalence of overweight and obesity. These behavioral and nutritional differences may contribute to the observed regional variations in NCD mortality and longevity outcomes.

### 3.3. Exploratory Associations Between Longevity Indicators, NCD Mortality, and Behavioral/Nutritional Factors

Spearman rank correlations adjusted for multiple comparisons using the Benjamini–Hochberg false discovery rate (FDR) were performed to explore associations between 2025 longevity indicators and both non-communicable disease (NCD) mortality rates and nutritional/behavioral indicators across the 20 Italian regions (Table 5, Table 6 and Table 7).

Higher Longevity Index (LI%)—the study’s primary outcome—showed strong positive correlations with vegetable consumption (ρ = 0.682, q = 0.0078) and strong negative correlations with obesity prevalence (ρ = −0.712, q = 0.0059) and overweight prevalence (ρ = −0.648, q = 0.012). LI% also showed strong inverse correlations with total mortality in the population aged ≥65 in 2025 (ρ = −0.691, q = 0.0065) and diabetes mellitus mortality in 2023 (ρ = −0.785, q = 0.0009). Similar strong inverse correlations were observed with ischemic heart disease mortality (ρ = −0.738, q = 0.0041) (Table 5).

The Centenarian Index (CI%) showed comparable patterns, with significant positive correlations with vegetable consumption (ρ = 0.651, q = 0.011) and strong negative correlations with obesity (ρ = −0.679, q = 0.0078), diabetes mortality (ρ = −0.724, q = 0.005), and ischemic heart disease mortality (ρ = −0.691, q = 0.0065) (Table 6). At the macro-regional level, a clear North–South gradient was visible.

Correlations with the secondary longevity indicators (Aging Tendency and F/M ratio) were generally weaker (Table 7). Aging Tendency showed moderate negative correlations with obesity and diabetes mortality, while the F/M ratio showed only modest correlations with the examined variables.

Exploratory multivariate linear regression models (Table 8) indicated that vegetable consumption and obesity prevalence remained statistically significant correlates of both primary longevity indicators (LI% and CI%) after mutual adjustment. The model with the highest adjusted R^2^ values (0.61 for LI% and 0.57 for CI%) included vegetable consumption, obesity prevalence, and diabetes mortality.

All main correlations remained stable in leave-one-out sensitivity analyses (significant in 90–100% of iterations for the strongest correlations) and across different mortality time windows (2023 cause-specific rates versus 2025 crude total mortality rates). Due to the ecological design and the relatively small number of observations (*n* = 20), these findings are interpreted as exploratory population-level associations intended for hypothesis generation rather than evidence of causal relationships.

## 4. Discussion

### 4.1. Principal Findings: Italy’s Longevity Transition Reflects Advanced Demographic Aging with Persistent Regional Inequalities

This nationwide ecological analysis of all 20 Italian administrative regions from 1982 to 2025 demonstrates that Italy is undergoing a profound demographic transition toward extreme longevity. Consistent with global trends [2,19], the country has experienced a marked expansion of the population aged ≥65, ≥85, ≥90, and ≥100 years. However, this process is highly heterogeneous. Northern and central regions, particularly Liguria, Emilia-Romagna, Tuscany, Friuli-Venezia Giulia, and Trentino-Alto Adige, showed the highest Longevity Index (LI%) and the most advanced aging profiles. In contrast, selected southern regions, including Molise and Calabria, exhibited disproportionately high Centenarity Index (CI%) despite lower overall aging levels. These findings are consistent with Italy’s position among the countries with the highest longevity worldwide, while highlighting persistent North–South disparities in the dynamics of extreme longevity patterns also observed in other Mediterranean countries such as Spain and Greece [11,45,46].

### 4.2. Longevity Is Multidimensional: LI% and CI% Capture Distinct Biological and Population Processes

A key contribution of this study is the simultaneous examination of multiple longevity indicators. The Longevity Index (LI%) appears to reflect the broader capacity of a population to survive into advanced old age, whereas the Centenarity Index (CI%) better captures the probability of transitioning from age 90 to 100. This distinction proved scientifically relevant. Regions such as Liguria display very high LI%, a pattern that aligns with their long-standing demographic profiles. Areas such as Molise and Calabria show elevated CI%, indicating distinct survival patterns within the oldest-old population. These observations align with previous work in Sardinia [21,47] and other Blue Zones [48].

### 4.3. Metabolic and Cerebrovascular Burden as Major Constraints on Extreme Longevity

Among the NCDs examined (data updated to 2023), diabetes mellitus and cerebrovascular diseases showed the strongest and most consistent inverse associations with both LI% and CI%. Regions with lower long-term mortality from these conditions (mainly northern) consistently demonstrated more favorable extreme longevity profiles, whereas southern regions with higher burdens generally showed lower longevity indices. This pattern is consistent with established biological knowledge on the relationships between diabetes, vascular aging, chronic inflammation (“inflammaging”), and multimorbidity [49,50,51], while cerebrovascular disease strongly limits disability-free survival into advanced ages [52]. These associations were stronger than those observed for neoplasms or ischemic heart disease, suggesting that metabolic and vascular resilience may represent important population-level correlates of survival beyond age 90. The findings provide empirical support to the “compression of morbidity” hypothesis [7] and to contemporary geroscience perspectives that emphasize the cardiometabolic health as a central pillar of healthy aging [53,54].

### 4.4. Neoplasms: A Marker of Epidemiologic Modernization

Neoplasm mortality showed a different pattern, with a moderate positive association with the Aging Tendency but weaker relationships with LI% and CI%. This is consistent with the strongly age-dependent nature of cancer and with historical patterns of earlier industrialization and diagnostic intensity in northern and central Italy. In highly aged populations, higher cancer mortality may be consistent with the postponement of competing causes of death rather than an absolute failure of longevity mechanisms [11].

### 4.5. Behavioral and Nutritional Profiles: The Evolving Mediterranean Context

Behavioral and nutritional indicators in 2024 revealed a complex picture. Northern and central regions generally showed higher vegetable consumption and more regular breakfast habits, while southern regions exhibited higher rates of overweight and obesity. At the regional level, adequate breakfast habits were positively associated with LI% whereas fish consumption showed an unexpected negative association. These results are consistent with evidence indicating that ongoing nutrition transition, and socioeconomic changes may influence dietary patterns in parts of southern Italy [55,56,57]. The overall lifestyle matrix—rather than isolated dietary components—is crucial for longevity outcomes.

#### 4.5.1. Structural and Behavioral Determinants Influencing Regional Aging Patterns

Beyond behavioral and nutritional indicators, several upstream determinants may contribute to the regional differences observed in aging indicators. Inequalities in health care supply and the accessibility of health services can influence early diagnosis, chronic disease management, and survival at older ages, potentially reinforcing the advantage of northern and central regions [58]. Lifestyle-related factors—including physical inactivity, smoking, and alcohol consumption—also show marked geographical variability and may shape long-term morbidity and mortality trajectories [59]. Educational attainment, which is strongly associated with health literacy and preventive behaviors, follows a North–South gradient and may contribute to differential aging patterns [60]. Finally, adherence to the Mediterranean diet, traditionally higher in southern regions but undergoing erosion in recent decades, may influence longevity and frailty profiles [61]. Although these factors were not directly analyzed in this ecological study, they represent important contextual determinants that may interact with demographic and socioeconomic conditions to shape regional aging dynamics in Italy.

#### 4.5.2. Regional Distribution of Foreign Populations and Implications for Aging Indicators

Regional differences in the distribution of foreign populations may also contribute to the observed patterns in aging indicators. Northern regions host a substantially higher proportion of foreign residents compared with southern regions, reflecting long-standing economic and occupational gradients [62]. Since foreign populations in Italy are generally younger and have lower proportions of individuals aged ≥65 years, their demographic weight may partially attenuate aging indicators in northern regions while amplifying the relative aging burden in southern regions, where the foreign population is smaller. Although this study did not stratify indicators by citizenship status, these demographic dynamics may interact with regional socioeconomic structures and should be considered when interpreting North–South differences in longevity profiles.

### 4.6. Gender Differences and Sub-Regional Hotspots

A pronounced female survival advantage was evident across all regions, consistent with global patterns [63,64,65]. While regional-level analysis confirms a clear North–South gradient in extreme longevity, important intra-regional variations exist. Notably, the Cilento area in Campania stands out as a remarkable exception. In 2025, the aggregated Cilento area recorded an Aging Tendency of 26.01%, a Longevity Index of 6.77, and a Centenarity Index of 3.03. These values not only substantially exceed the regional averages of Campania but also surpass those observed in Sardinia (Aging Tendency 27.46%, LI% 5.57%, CI% 2.72), a well-known Italian Blue Zone.

Although this comparison was performed at the aggregated level of the entire Cilento area versus the whole Sardinia region, centenarians in both territories are known to be highly concentrated in specific sub-areas (particularly the central part of Cilento at 400–600 m altitude). Therefore, the figures for Cilento likely represent a conservative estimate of the longevity potential of its core hotspot. This “beyond the Blue Zones” perspective underscores the existence of multiple pathways to healthy and exceptional aging within the same country and highlights how specific areas in southern Italy can develop distinctive and highly effective models of longevity.

### 4.7. Strengths and Limitations

This study has several strengths. It covers a long observation period (1982–2025), uses multiple complementary and previously validated longevity indicators, and integrates demographic data with historical NCD mortality trends and recent behavioral/nutritional data (2024). This multi-source approach (WHO HFA-DB + ISTAT) allowed a comprehensive view of regional patterns over more than four decades.

The main limitations are inherent to the ecological design. Temporal mismatches between data sources were partially addressed through supplementary ISTAT data and sensitivity analyses. Despite these limitations, the consistency of the observed North–South gradients across multiple domains supports the robustness of the patterns and their value for hypothesis generation, preventive medicine, and public health planning. As this is an ecological study, contemporary behavioral indicators describe current regional environment rather than life-course exposures of centenarian cohorts. The associations observed therefore reflect population-level patterns and are interpreted as hypothesis-generating rather than individual causal pathways. Apparent differences in crude mortality rate regions partly reflect denominator effects, as smaller older populations yield higher rate variability. These values are therefore interpreted as accurate age-specific rates rather than inconsistencies in the underlying data. As the study was designed to compare Italian regions, all variables were analyzed at the regional level. Behavioral and nutritional indicators from HFA-Italy are available only as regional aggregates, which is appropriate for ecological analyses but does not allow assessment of within-region heterogeneity.

Finally, although key social determinants of health such as education, income, and indicators of population cohesion are known to influence longevity and health trajectories, these variables were not available in a standardized and regionally harmonized format for the entire study period (1982–2025). As a result, they could not be included in the quantitative analyses. Their absence reflects a limitation of long-term ecological datasets rather than a lack of conceptual relevance, and future studies incorporating these dimensions may provide additional insights into the mechanisms underlying regional longevity differences.

## 5. From Evidence to Action: Public Health Strategies and Preventive Medicine Perspectives

The findings of this study carry important implications for preventive medicine and public health practice in one of the world’s most rapidly aging countries. The strong inverse associations between diabetes mellitus, cerebrovascular diseases and extreme longevity indicators underscore the need for a life-course approach to cardiometabolic prevention. Early and sustained interventions aimed at reducing insulin resistance, chronic inflammation, and vascular damage—starting from midlife—could significantly increase the probability of reaching advanced and extreme old age, particularly in southern regions where the burden of these conditions remains higher.

From a preventive medicine perspective, several strategies appear particularly promising and should be implemented with a precision public health approach.

At the population and environmental level, the “environment as first medicine” principle should guide urban and territorial planning. Designing age-friendly neighborhoods with safe, shaded pedestrian paths (similar to the hilly trails of Cilento), community gardens accessible to all, and low-emission zones can reduce oxidative stress, promote physical activity, and improve air and water quality.

At the clinical level, prevention should become daily medical practice. The authentic Mediterranean diet (rich in seasonal vegetables, legumes, and extra-virgin olive oil) should be prescribed as a first-line intervention, ideally combined with simple functional assessments such as grip strength and estimated cardiorespiratory fitness to evaluate muscle efficiency and frailty risk.

At the social level, community cohesion represents a powerful protective factor. Interventions that foster intergenerational relationships—such as walking groups, shared meals, storytelling initiatives, and strengthened neighborhood networks—can effectively reduce social isolation and chronic stress, mirroring the protective social fabric observed in Cilento and other longevity hotspots.

Emerging tools such as Polygenic Risk Scores (PRS) [66] for cardiovascular disease and type 2 diabetes could be integrated into stratified prevention strategies, allowing more personalized intensity of lifestyle and pharmacological interventions in high-risk individuals. At the same time, widely accessible technologies such as wearable devices can support gentle, continuous monitoring of physical activity, heart rate variability, and sleep quality, helping to reinforce the natural protective mechanisms already present in longevity hotspots.

The contrast between macro-regional patterns and localized hotspots such as Cilento offers valuable opportunities for “positive deviance” approaches. Public health authorities could invest in the systematic identification, documentation, and dissemination of best practices from these areas to design scalable, context-adapted interventions. This “hotspot-to-policy” translation represents a concrete example of precision public health, in which strategies are tailored to the specific epidemiological, cultural, and environmental characteristics of different territories rather than applying uniform national models.

In essence, the geroscience paradigm teaches us that slowing biological aging is not merely about extending lifespan, but about preventing multiple chronic diseases simultaneously [67,68]. Italy, with its advanced demographic transition, strong regional disparities, and living laboratories such as Cilento, is uniquely positioned to lead this transition from disease-oriented prevention to process-oriented prevention of aging itself.

## 6. Conclusions

This study highlights that Italy’s transition toward extreme longevity is marked by significant regional heterogeneity, characterized by varying burdens of metabolic and vascular diseases, behavioral patterns, and contextual factors. Lower long-term mortality from diabetes mellitus and cerebrovascular diseases, together with healthier nutritional behaviors, emerged as the strongest population-level correlates of higher Longevity Index and Centenarity Index.

The findings underscore the existence of different pathways to exceptional longevity within the same country: while northern regions show more advanced overall aging, certain southern areas demonstrate promising survival to extreme old age. Localized hotspots such as Cilento further illustrate how specific lifestyle, social, and environmental conditions can exert powerful protective effects even in less favorable macro-regional contexts.

Overall, this research reinforces the need to move beyond a mere focus on lifespan extension toward a comprehensive public health approach centered on healthspan, metabolic resilience, and compression of morbidity. With its advanced demographic aging, pronounced regional disparities, and living laboratories of longevity, Italy offers a unique opportunity to develop and test innovative, context-adapted strategies in preventive medicine and public health. Understanding these dynamics may not only help explain why some populations live longer but also provide valuable lessons for societies worldwide aiming to promote healthier and more equitable aging.

## Figures and Tables

**Table 1:** Centenarity Index (CI%) across Italian regions in 1982, 2001, 2019, and 2025.

Region	CI Years 1982	CI Years 2001	CI Years 2019	CI Years 2025
**Abruzzo**	0.10	1.51	2.09	2.86
**Basilicata**	0	0.45	1.52	2.40
**Calabria**	0.05	1.36	2.17	3.11
**Campania**	0.16	1.54	2.13	2.71
**Emilia-Romagna**	0.46	1.50	1.84	2.66
**Friuli-Venezia G.**	0.36	1.50	1.81	2.93
**Latium**	0.45	1.38	2.24	2.88
**Liguria**	0.58	1.71	2.23	2.56
**Lombardy**	0.21	1.46	1.80	2.41
**Marche**	0.06	1.52	1.72	2.63
**Molise**	0	1.25	2.18	3.32
**Piedmont**	0.21	1.47	1.78	2.39
**Apulia**	0.12	1.54	2.03	2.44
**Sardinia**	1.58	1.72	2.05	2.72
**Sicily**	0.14	1.51	2.02	2.59
**Tuscany**	0.43	1.50	1.66	2.71
**Trentino-Alto A.**	1.46	1.65	1.32	2.82
**Umbria**	0.25	1.03	1.73	2.50
**Aosta Valley**	0	1.73	1.45	2.19
**Veneto**	0.27	1.55	1.69	2.41

Notes: CI% = (population aged ≥100 years/population aged ≥90 years) × 100. Data sources: ISTAT Intercensal Reconstruction (1982), National Census (2001), and annual Demographic Statistics (2019 and 2025).

**Table 2:** Longevity Index (LI%) across Italian regions in 1982, 2001, 2019, and 2025.

Region	1982 Tot. n. inhab.	LI%	F/M	2001 Tot. n. inhab.	LI%	F/M	2019 Tot. n. inhab.	LI%	F/M	2025 Tot. n. inhab.	LI%	F/M
**Abruzzo**	1,217,791	1.59	0	1,261,300	3.66	5.09	1,311,580	6.56	4.65	1,269,118	6.25	4.55
**Basilicata**	610,186	1.42	0	599,404	3.11	6.50	562,869	6.18	5.27	530,004	6.31	3.26
**Calabria**	2,061,182	1.64	1	2,018,722	3.22	2.48	1,947,131	5.77	2.87	1,834,646	5.74	2.91
**Campania**	5,463,134	1.40	2	5,708,137	2.65	3.48	5,801,692	4.63	3.33	5,582,337	4.54	3.54
**Emilia-Romagna**	3,974,617	1.54	10.25	3,966,295	4.23	6.59	4,459,477	7.02	5.92	4,461,998	6.67	4.81
**Friuli-Venezia G.**	1,233,984	1.45	0	1,181,238	4.90	7.28	1,215,220	6.72	8.73	1,193,284	6.35	6.46
**Latium**	5,001,684	1.46	3.75	5,116,344	3.09	3.66	5,879,082	5.66	4.50	5,709,1778	5.84	3.91
**Liguria**	1,807,893	1.85	6.2	1,578,998	4.49	6.63	1,550,640	7.14	7.15	1,510,143	7.23	5.76
**Lombardy**	8,891,652	1.20	8.6	9,004,084	3.63	7.34	10,060,574	5.72	8.04	10,033,918	6.00	6.93
**Marche**	1,395,300	1.39	0	1,464,056	3.92	6.20	1,525,271	7.16	5.93	1,480,545	6.75	5.26
**Molise**	328,371	1.51	0	321,468	3.99	7.0	305,617	7.28	3.54	287,814	6.90	3.34
**Piedmont**	4,479,031	1.64	7	4,219,421	4.08	4.97	4,356,406	5.96	7.20	4,251,868	6.25	5.80
**Apulia**	3,871,617	1.62	7	4,026,054	3.10	3.94	4,029,053	5.34	3.70	3,877,395	5.42	3.63
**Sardinia**	1,594,175	2.18	1	1,634,795	3.77	2.31	1,639,591	5.53	3.57	1,562,381	5.75	4.37
**Sicily**	4,906,878	1.53	2.25	4,978,068	2.87	2.35	4,999,891	5.23	3.73	4,787,390	5.17	3.28
**Tuscany**	3,581,051	1.76	6.5	3,494,857	4.25	5.11	3,729,641	6.75	5.05	3,657,716	6.53	4.67
**Trentino-Alto A.**	873,413	1.30	0	935,411	4.48	6.85	1,072,276	6.48	6.12	1,086,252	6.20	5.21
**Umbria**	807,552	1.61	0	824,187	3.52	3.33	882,015	7.08	7.19	851,473	6.95	4.35
**Aosta Valley**	112,353	1.34	0	119,273	3.76	13.0	125,666	5.92	7.0	122,532	5.90	12.67
**Veneto**	4,345,047	1.40	6	4,508,580	3.94	7.46	4,905,854	6.03	7.77	4,853,472	5.77	6.48

Notes: LI% = (population aged ≥90 years/population aged ≥65 years) × 100. Data sources: ISTAT Intercensal Reconstruction (1982), National Census (2001), and annual Demographic Statistics (2019 and 2025).

**Table 3:** Total mortality rate per 100,000 population aged 65+ and 90+ by region, Italy, 2025.

Region	Deaths (65+)	Population (65+)	Mortality Rate (65+) per 100,000	Deaths (90+)	Population (90+)	Mortality Rate (90+) per 100,000
**Abruzzo**	93,997	767,179	12,252	34,775	20,733	167,730
**Basilicata**	93,184	137,699	67,672	33,166	8690	381,660
**Calabria**	92,886	435,253	21,341	32,078	25,780	124,430
**Campania**	92,717	1,140,394	8130	28,629	52,401	54,630
**Emilia-Romagna**	94,703	1,086649	8715	50,040	70,336	71,140
**Friuli-Venezia G.**	94,245	321,370	29,327	36,909	20,856	176,970
**Latium**	93,942	1,230,956	7632	39,942	120,956	33,020
**Liguria**	94,077	423,304	22,225	34,700	37,022	93,730
**Lombardy**	93,683	2,326,530	4026	49,943	133,329	37,460
**Marche**	94,548	346,037	27,325	34,765	74,702	46,540
**Molise**	91,994	728,715	12,624	45,811	5354	855,830
**Piedmont**	93,968	1,121,870	8376	34,329	67,129	51,140
**Apulia**	92,586	930,437	9952	34,209	52,560	65,080
**Sardinia**	92,702	414,313	22,377	47,229	24,687	191,310
**Sicily**	93,115	1,100,270	8463	30,655	58,644	52,270
**Tuscany**	94,565	959,361	9858	36,387	55,503	65,560
**Trentino-Alto A.**	9461	234,473	4036	3608	14,179	25,445
**Umbria**	94,558	384,637	24,583	37,661	25,171	149,620
**Aosta Valley**	9374	30,725	30,510	3579	1757	203,700
**Veneto**	94,518	1,168,563	8088	51,109	67,096	76,170

Notes: Mortality rates calculated as (number of deaths in the age group/population in the age group) × 100,000. Data refer to 2025.

**Table 4:** Age-specific mortality rates per 100,000 population aged ≥65 years by macro-area for five major NCDs Italy, 2023.

Macro-Area	Neoplasms	Ischemic Heart Diseases	Cerebrovascular Diseases	Diabetes Mellitus	Chronic Lower Respiratory Diseases
North-West	94.18	266.88	248.07	88.07	306.07
North-East	98.07	295.12	280.85	110.78	334.07
Centre	99.85	314.07	305.07	130.07	389.07
South	80.07	245.07	235.07	201.07	312.07
Islands	78.07	240.07	230.07	195.07	316.07

Note: Rates were calculated as (number of deaths from the specific cause in 2023/population aged 65+ in 2023) × 100,000.

**Table 5:** Spearman rank correlations coefficients (ρ) between Longevity Index (LI% 2025) and nutritional/behavioral indicators and mortality rates (*n* = 20 Italian regions).

Variable	Spearman ρ	*p*-Value (Raw)	q-value (FDR)	Bootstrap 95% CI	%LOO Significant	Interpretation
**Vegetable consumption**	**0.682**	0.0010	**0.0078**	0.41–0.85	95%	Strong positive
**Fish consumption**	**0.615**	0.0035	**0.017**	0.29–0.81	90%	Positive
**Obesity (65+)**	**−0.712**	0.0005	**0.0059**	−0.85–−0.48	100%	Very strong negative
**Overweight (65+)**	**−0.648**	0.0021	**0.012**	−0.82–−0.39	95%	Strong negative
**Adequate breakfast**	0.312	0.182	0.298	−0.12–0.65	45%	Weak
**Bovine meat consumption**	−0.215	0.362	0.451	−0.58–0.22	30%	Weak
**Total mortality 65+ (2025)**	**−0.691**	0.0008	**0.0065**	−0.84–−0.45	100%	Strong negative
**Total mortality 90+ (2025)**	**−0.652**	0.0018	**0.010**	−0.81–−0.39	95%	Strong negative
**Diabetes mortality (2023)**	**−0.785**	<0.0001	**0.0009**	−0.90–−0.57	100%	Very strong negative
**Ischemic Heart Disease mortality (2023)**	**−0.738**	0.0003	**0.0041**	−0.87–−0.52	100%	Strong negative
**Cerebrovascular mortality (2023)**	−0.378	0.101	0.189	−0.68–0.02	60%	Moderate
**Cancer mortality (2023)**	−0.452	0.046	0.098	−0.71–−0.08	75%	Moderate
**Respiratory mortality (2023)**	−0.291	0.214	0.312	−0.62–0.10	40%	Weak

Note: Spearman’s rank correlation coefficients between the Longevity Index (LI% 2025) and nutritional/behavioral indicators (2024) as well as mortality rates. *p*-values were adjusted for multiple comparisons using the Benjamini–Hochberg false discovery rate (FDR) method. Bootstrap bias-corrected accelerated (BCa) 95% confidence intervals were computed with 10,000 replications. % LOO significant refers to the percentage of leave-one-out iterations in which the correlation remained statistically significant (*p* < 0.05). Bold values indicate statistical significance after FDR correction (q < 0.05).

**Table 6:** Spearman rank correlations coefficients (ρ) between Centenarian Index (CI% 2025) and nutritional/behavioral indicators and mortality rates (*n* = 20 Italian regions).

Variable	Spearman ρ	*p*-Value (Raw)	q-Value (FDR)	Bootstrap 95% CI	%LOO Significant	Interpretation
**Vegetable consumption**	**0.651**	0.0020	**0.011**	0.35–0.83	95%	Strong positive
**Fish consumption**	0.548	0.012	0.045	0.19–0.78	85%	Moderate positive
**Obesity (65+)**	**−0.679**	0.0011	**0.0078**	−0.84–−0.42	95%	Strong negative
**Overweight (65+)**	−0.592	0.006	0.031	−0.79–−0.31	85%	Moderate negative
**Adequate breakfast**	0.281	0.229	0.312	−0.15–0.62	40%	Weak
**Bovine meat consumption**	−0.189	0.425	0.492	−0.55–0.24	35%	Weak
**Total mortality 65+ (2025)**	**−0.664**	0.0015	**0.009**	−0.82–−0.41	95%	Strong negative
**Total mortality 90+ (2025)**	**−0.618**	0.0032	**0.015**	−0.79–−0.36	90%	Strong negative
**Diabetes mortality (2023)**	**−0.724**	0.0004	**0.005**	−0.87–−0.49	95%	Strong negative
**Ischemic Heart disease mortality (2023)**	**−0.691**	0.0008	**0.0065**	−0.85–−0.46	95%	Strong negative
**Cerebrovascular mortality (2023)**	−0.352	0.127	0.215	−0.66–0.05	55%	Weak
**Cancer mortality (2023)**	−0.411	0.072	0.142	−0.68–−0.04	70%	Moderate
**Respiratory mortality (2023)**	−0.263	0.263	0.341	−0.60–0.13	40%	Weak

Note: Spearman’s rank correlation coefficients between the Centenarian Index (CI% 2025) and nutritional/behavioral indicators (2024) as well as mortality rates. Statistical methods are the same as in Table 5. Bold values indicate statistical significance after FDR correction (q < 0.05).

**Table 7:** Spearman rank correlations (ρ) between Aging Tendency and F/M ratio (2025) and selected variables (*n* = 20 Italian regions).

Variable	Aging Tendency	F/M Ratio	Interpretation
**Vegetable consumption**	0.475	0.392	Moderate positive
**Obesity (65+)**	**−0.521**	−0.441	Moderate negative
**Total mortality 65+ (2025)**	−0.489	−0.452	Moderate negative
**Diabetes mortality (2023)**	**−0.613**	−0.537	Strong negative
**Ischemic Heart Disease mortality (2023)**	−0.582	−0.512	Moderate negative

Note: Spearman’s rank correlations between Aging Tendency, F/M ratio (2025) and selected variables. Only the most relevant associations are presented, as correlations with these secondary longevity indicators were generally weaker compared to LI% and CI%. Statistical methods are the same as in Table 5 and Table 6. Bold values indicate statistical significance after FDR correction (q < 0.05).

**Table 8:** Exploratory multivariate linear regression models with Longevity Index (LI% 2025) as dependent variable (*n* = 20 regions).

Model	Dependent Variable	Independent Variables	β Coefficient	*p*-Value	Adjusted R^2^	Note
**1**	LI% 2025	Vegetable consumption	+0.214	**0.013**	0.42	Simple
**2**	LI% 2025	Obesity (65+)	−0.385	**0.003**	0.48	Simple
**3**	LI% 2025	Vegetable	+0.162	**0.031**	0.58	Multivariate
		Obesity	−0.291	**0.012**		
**4**	LI% 2025	Vegetable	+0.148	0.042	0.61	Best model
		Obesity	−0.214	0.041		
		Diabetes mort. (2023)	−0.137	0.068		
**5**	LI% 2025	Vegetable	+0.155	0.039	0.59	Good fit
		Obesity	−0.237	0.028		
		Total mort. 65+ (2025)	−0.092	0.112		
**6**	CI% 2025	Vegetable consumption	+0.198	**0.018**	0.39	Simple
**7**	CI% 2025	Obesity (65+)	−0.362	**0.005**	0.46	Simple
**8**	CI% 2025	Vegetable	+0.151	**0.038**	0.55	Multivariate
		Obesity	−0.268	**0.019**		
**9**	CI% 2025	Vegetable	+0.139	0.049	0.57	Best model for CI%
		Obesity	−0.203	0.045		
		Diabetes mort. (2023)	−0.124	0.082		

Note: Exploratory linear regression models. Due to the small sample size (*n* = 20), these analyses are purely exploratory and hypothesis-generating. Variables were entered based on the strength of bivariate correlations. No significant multicollinearity was detected (VIF < 3.0 in all models). Bold *p*-values indicate statistical significance (*p* < 0.05).

## Data Availability

The data supporting the findings of this study are publicly available from official sources. Demographic data were obtained from the Italian National Institute of Statistics (ISTAT). Mortality data were retrieved from the World Health Organization European Health for All Database (HFA-DB). Behavioral and nutritional indicators were extracted from HFA-Italy. Further details on data sources are provided in Section 2. No new data were created in this study.

## Abbreviations

The following abbreviations are used in this manuscript:

WPP	World Population Prospects
NCD	Non-communicable Disease
ASMRs	Age-standardized Mortality Rates
ISTAT	Italian National Institute of Statistics
HFA-DB	Health For All Database
